# Shared Genetic Risk in the Association of Screen Time With Psychiatric Problems in Children

**DOI:** 10.1001/jamanetworkopen.2023.41502

**Published:** 2023-11-06

**Authors:** Yingzhe Zhang, Karmel W. Choi, Scott W. Delaney, Tian Ge, Jean-Baptiste Pingault, Henning Tiemeier

**Affiliations:** 1 Department of Epidemiology, Harvard T.H. Chan School of Public Health, Boston, Massachusetts; 2 Center for Precision Psychiatry, Department of Psychiatry, Massachusetts General Hospital, Boston; 3 Psychiatric and Neurodevelopmental Genetics Unit, Center for Genomic Medicine, Massachusetts General Hospital, Boston; 4 Department of Environmental Health, Harvard T.H. Chan School of Public Health, Boston, Massachusetts; 5 Department of Clinical, Educational and Health Psychology, University College London, London, United Kingdom; 6 Social, Genetic, and Developmental Psychiatry Centre, King’s College London, London, United Kingdom; 7 Department of Social and Behavioral Sciences, Harvard T.H. Chan School of Public Health, Boston, Massachusetts

## Abstract

**Importance:**

Children’s exposure to screen time has been associated with poor mental health outcomes, yet the role of genetic factors remains largely unknown.

**Objective:**

To assess the extent of genetic confounding in the associations between screen time and attention problems or internalizing problems in preadolescent children.

**Design, Setting, and Participants:**

This cohort study analyzed data obtained between 2016 and 2019 from the Adolescent Brain Cognitive Development Study at 21 sites in the US. The sample included children aged 9 to 11 years of genetically assigned European ancestry with self-reported screen time. Data were analyzed between November 2021 and September 2023.

**Exposure:**

Child-reported daily screen time (in hours) was ascertained from questionnaires completed by the children at baseline.

**Main Outcomes and Measures:**

Child psychiatric problems, specifically attention and internalizing problems, were measured with the parent-completed Achenbach Child Behavior Checklist at the 1-year follow-up. Genetic sensitivity analyses model (Gsens) was used, which incorporated polygenic risk scores (PRSs) of both exposure and outcomes as well as either single-nucleotide variant (SNV; formerly single-nucleotide polymorphism)–based heritability or twin-based heritability to estimate genetic confounding.

**Results:**

The 4262 children in the sample included 2269 males (53.2%) with a mean (SD) age of 9.9 (0.6) years. Child screen time was associated with attention problems (β = 0.10 SD; 95% CI, 0.07-0.13 SD) and internalizing problems (β = 0.03 SD; 95% CI, 0.003-0.06 SD). The television time PRS was associated with child screen time (β = 0.18 SD; 95% CI, 0.14-0.23 SD), the attention-deficit/hyperactivity disorder PRS was associated with attention problems (β = 0.13 SD; 95% CI, 0.10-0.16 SD), and the depression PRS was associated with internalizing problems (β = 0.10 SD; 95% CI, 0.07-0.13 SD). These PRSs were associated with cross-traits, suggesting genetic confounding. Estimates using PRSs and SNV-based heritability showed that genetic confounding accounted for most of the association between child screen time and attention problems and for 42.7% of the association between child screen time and internalizing problems. When PRSs and twin-based heritability estimates were used, genetic confounding fully explained both associations.

**Conclusions and Relevance:**

Results of this study suggest that genetic confounding may explain a substantial part of the associations between child screen time and psychiatric problems. Genetic confounding should be considered in sociobehavioral studies of modifiable factors for youth mental health.

## Key Points

**Question:**

To what extent can genetic variations explain the associations between screen time and psychiatric problems in children?

**Findings:**

In this cohort study of 4262 pediatric participants in the Adolescent Brain Cognitive Development Study, genetic confounding accounted for much of the association between screen time and attention problems and part of the association with internalizing problems in the model using molecular-based heritability. In models using twin-based heritability, genetic confounding fully explained both associations.

**Meaning:**

Findings of this study suggest that genetic confounding should be considered in sociobehavioral studies of modifiable factors for youth mental health.

## Teaser

This cohort study evaluates the extent of genetic confounding in the association of screen time with mental health problems among preadolescent children.

## Introduction

The potential adverse association between screen time and child mental health are widely discussed. A recent meta-analysis of 87 studies found that longer duration of screen time was associated with small increases in child internalizing problems (*r* = 0.07).^1^ Another study suggested that screen time was associated with more childhood attention problems.^2^ Similarly, a large-scale population-based study in the US reported that screen time of more than 4 hours per day was associated with increased mood and attention problems among children and adolescents.^3^ Although some inconsistent results have emerged,^4,5,6,7,8^ excessive screen time is widely recognized to be associated with child psychiatric problems.

Although phenotypic associations between screen time and psychiatric problems have been widely studied, the potential role of genetics in these associations remains largely unknown. Child psychiatric problems, including attention and internalizing problems, are affected by genetics.^9,10^ Scientists have begun to investigate how genes play a role in behavioral traits, suggesting that genetic variants could affect screen time through neurodevelopmental pathways by altering central nervous system genetic expression.^11,12^ Shared genetic risk factors (pleiotropy) are common and of relevance to many genetic variants identified in behavioral genetic research.^13,14^ Ignoring genetic associations with behavioral traits may bias research examining environmental outcomes. As both screen time and psychiatric problems could be directly affected by genetic factors, an individual genotype as a common cause (ie, genetic confounding) may generate noncausal associations between child screen time and psychiatric problems.^15,16^ Given the interest in improving child well-being by reducing screen time,^17^ evaluating potential genetic confounding to better identify the causal association between screen time and child psychiatric problems has public health implications.

**Figure 1.**
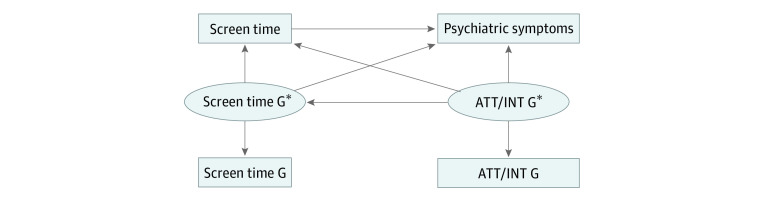
Genetic Confounding Sensitivity Analysis Framework G refers to an underlying genetic factor, including measurement error. G* refers to the latent variable that captures the heritability of the corresponded trait. ATT indicates attention problems; INT, internalizing problems.

Prior studies considering genetic confounding in other contexts have often adjusted their analyses for polygenic risk score (PRS).^18,19^ Overall, PRS is a weighted sum score indicating the risk of certain disease due to individual genotype. It has been widely used in prediction studies but rarely in causation studies. Because PRS is based on imperfectly measured additive properties of common variants, it can be construed as a noisy measure of heritability that generally explains little variance of any given psychiatric trait.^20,21^ Thus, adjusting models for PRSs likely underestimates the confounding arising from genetic factors. To correct the measurement error of using PRS to represent a genetic factor, Pingault et al^22^ proposed a genetic sensitivity analysis method called Gsens, which uses information from both PRSs and more comprehensive heritability estimates. In this framework, the overall phenotypic associations are divided into (1) genetic confounding and (2) the residual association excluding genetic confounding. Specifically, in the Gsens model, either single-nucleotide variant (SNV; formerly single-nucleotide polymorphism [SNP])–based heritability or twin-based heritability estimates can be used in structural equation models (Figure 1) to account for possible PRS measurement error. Genetic confounding is different from genetic correlation, which includes both genetic confounding and mediated pleiotropy.

In the Gsens model (Figure 1), the polygenic score G measures the underlying genetic factor, which includes measurement error. The latent variable G* captures the heritability of the corresponding trait under the SNV-based heritability or twin-based heritability scenario. The SNV-based heritability captures common genetic variations but not rare variants,^23^ insertions, or deletions that may partially account for the heritability of attention-deficit/hyperactivity disorder (ADHD) and depression.^24,25^ Thus, analyses using SNV-based heritability provide a lower (ie, possibly underestimated) bound of genetic confounding. In contrast, twin-based heritability may overestimate heritability due to genetic interactions,^26^ gene-environment interactions,^27^ or a violated equal-environment assumption in twin studies.^28^ Thus, analyses using twin-based heritability provide an upper-bound estimate of genetic confounding. Hence, the Gsens method provides a range for potential genetic confounding under either SNV-based heritability or twin-based heritability scenarios.

In this cohort study, using the Gsens model, we aimed to assess the extent of genetic confounding in the associations between child screen time and attention problems or internalizing problems in preadolescent children. We hypothesized that genetic factors combining both PRS and different heritability estimates would account for a substantial part of these associations.

## Methods

We used data from 2016 to 2019 from the Adolescent Brain Cognitive Development (ABCD) Study, release 5.0, of children aged 9 to 11 years participating at 21 study sites across the US.^29,30^ The ABCD Study is a prospective cohort that was designed to reflect the sociodemographic diversity of the US and that recruits primarily from schools. Because PRSs were derived from genome-wide association studies (GWASs) in European ancestry populations, we included only 4262 unrelated children with genetically identified European ancestry (eMethods in Supplement 1). Parents provided written informed consent, and all participating children assented. The institutional review board at each data collection site approved the research protocol.^30^ We followed the Strengthening the Reporting of Genetic Association Studies (STREGA) reporting guideline.

### Genotyping and PRS

Detailed information about genotyping in the ABCD Study is provided elsewhere.^31^ We used the ABCD Study release 4.0 genotype data. After quality control and imputation, we extracted 6 833 710 genetic variants (eMethods in Supplement 1).

To calculate PRSs, we chose large-sample GWASs of phenotypes corresponding with the ABCD Study child measures. We computed genome-wide PRSs using statistics from the following GWAS data: leisure television watching time (n = 365 236),^11^ ADHD (n = 55 374),^32^ and major depression (n = 500 199).^33^ We used the leisure television watching time GWAS because it was the most relevant proxy phenotype for overall screen time, despite technological and behavioral changes. The PRSs for all traits were computed using a bayesian scoring method (PRS-CS software; Python) that places a continuous shrinkage prior on effect sizes.^34^

### Screen Time and Psychiatric Problems in Children

Participating children completed the 14-question Screen Time Questionnaire at baseline, providing self-reported measures of daily screen time ranging from 0 to 4 or more hours (eMethods in Supplement 1). Parents or caregivers also completed a shorter version of the Screen Time Questionnaire about their children’s total screen use. The more complete child-reported screen time was used as the primary exposure to avoid shared-reporter bias.

Parents completed the Achenbach Child Behavior Checklist 6/18 (for ages 6-18 years) at 1-year follow-up.^35^ We assessed attention problems using the attention problem subscale (10 items; score range: 0-20) and internalizing problems using the combined anxious or depressed, somatic complaints, and withdrawn or depressed subscales (32 items; score range: 0-64); higher scores for each subscale indicate higher severity.

### Covariates and Heritability

Age, sex, and study site were included as confounders in the association between child screen time and psychiatric problems. Family income, highest parental educational level, and maternal psychopathological disorder were considered to be additional potential confounders (eMethods in Supplement 1). We adjusted the models for the top 10 principal components (derived from principal components analysis) to account for residual confounding by genetic ancestry.

We assessed SNV-based heritability of screen time, attention problems, and internalizing problems using the GCTA-GREML software,^36^ adjusting for age, sex, study site, and top 10 principal components. We used rank-based, normality-transformed screen time and log-transformed psychiatric problem scores to optimally normalize the distribution and meet the model assumption when estimating the heritability (eFigure 1 in Supplement 1). We calculated twin-based heritability using 216 pairs of European monozygotic twins and 333 pairs of dizygotic twins from the ABCD Study using identity by descent segments^37^ (eMethods in Supplement 1).

### Statistical Analysis

We calculated the descriptive statistics for participants and compared them with self-identified European participants without genetic data for a nonresponse analysis. In primary analyses, first we examined the associations between child-reported screen time and parent-reported child attention problems or internalizing problems using linear regressions. Second, we quantified associations of the PRSs (television time, ADHD, and depression) with screen time and psychiatric problems using linear regression.

Third, we used the Gsens framework to quantify genetic confounding for the associations between screen time and attention and internalizing problems. We fit 3 sets of structural equation models. The first set used PRSs for the exposure and outcomes to adjust for genetic confounding in a simplistic way. In the second set, we modeled both SNV-based heritability and the PRSs to produce a lower-bound estimate of genetic confounding. In the third set, we modeled both twin-based heritability and PRSs to delineate the upper bound of genetic confounding. These models used standardized correlations, adjusting for sex, age, study site, and principal components. Because the observed PRS for screen time was quite predictive, the models used genetic information from both the exposure and the outcomes (eTable 1 in Supplement 1). For comparison, we also fit the models using only PRSs for the outcomes as sensitivity analyses.

In another sensitivity analysis, we also adjusted the models for family income and highest parental educational level. Separately, to show consistency, we compared associations between screen time and psychiatric problems using screen time data reported by children vs parents. We explored how the magnitude of genetic confounding may vary given different subtypes of screen time and different estimates of heritability. For simulation, we conducted Gsens analyses across an SNV-based heritability range between 0.01 to 0.3 for psychiatric problems.^11^

In the analyses, all PRSs, child-reported and parent-reported screen time, and pediatric psychiatric problems were standardized to mean 0 and SD 1 to facilitate interpretation and comparisons. Under all heritability scenarios, the direction of the association of screen time with child psychiatric problems was constrained to be beneficial on the basis of main phenotypic associations (ie, the minimum association was null, rather than screen time being unlikely protective). We used R, version 4.0.3 (R Foundation for Statistical Computing), with 2-sided hypothesis tests’ significance set at .05 for all analyses. Data were analyzed between November 2021 and September 2023.

## Results

The sample included 4262 children, of whom 1993 were females (46.8%) and 2269 were males (53.2%) with a mean (SD) baseline age of 9.9 (0.6) years (eTable 2 in Supplement 1). Children reported a mean (SD) daily screen time of 3.2 (2.6) hours. Using a shorter questionnaire capturing only approximate screen use hours, parents reported a mean (SD) screen time of 1.2 (0.6) hours per day for their children. Mean (SD) scores at the 1-year follow-up were 2.9 (3.4) points for attention problems and 5.4 (5.6) points for internalizing problems. eTable 3 in Supplement 1 provides the nonresponse analysis of the genetic data.

### Heritability Estimates 

Using GCTA-GREML, we found that the covariate-adjusted SNV-based heritability estimates (SE) were 0.08 (0.08) for child-reported screen time and 0.06 (0.08) for parent-reported screen time (Table). The heritability estimates of screen subtypes are provided in eTable 4 in Supplement 1. The covariate-adjusted SNV-based heritability estimates (SE) were 0.18 (0.08) for attention problem scores and 0.07 (0.08) for internalizing problem scores. Based on 216 monozygotic and 333 dizygotic twin pairs, the twin-based heritability estimates (SE) were 0.58 (0.08) for child-reported screen time, 0.88 (0.06) for attention problems, and 0.48 (0.08) for internalizing problems. We did not use the twin-based heritability estimate for parent-reported screen time because many parents reported the same amount of time for each twin, leading to an underestimated twin-based heritability.

**Table.** Heritability Estimates and Corresponding Sample Sizes^a^

	SNV-based heritability				Twin-based heritability			
	No. of children in ABCD Study	Estimated β (SE) in ABCD Study	No. of children in literature	Estimated β in literature, source	No. of twins in ABCD Study	Estimated β (SE) in ABCD Study	No. of twins in literature	Estimated β in literature, source
Attention problems	4314	0.18 (0.08)	55 374	0.22, Demontis et al^32^ 2019^b^	214 MZ 333 DZ	0.88 (0.06)	17 026 MZ 42 488 DZ	0.88, Larsson et al^38^ 2014^b^
Internalizing problems		0.07 (0.08)	64 641	0.02, Jami et al^39^ 2022^c^; 0.09, Howard et al^33^ 2019^d^		0.48 (0.08)	4367^e^	0.42, Cheesman et al^40^ 2017^f^
Child-reported screen time	4303	0.08 (0.08)	422 218	0.16, van de Vegte et al^11^ 2020^g^	216 MZ 328 DZ	0.58 (0.08)	1961 MZ 3220 DZ	0.37, Ayorech et al^41^ 2017^h^
Parent-reported screen time	4273	0.06 (0.08)			NA	NA	NA	NA

Abbreviations: ABCD, Adolescent Brain Cognitive Development; DZ, dizygotic twins; MZ, monozygotic twins; NA, not applicable; SNV, single-nucleotide variant.

^a^ The parent-reported screen time values in twins were highly associated such that twin-based heritability cannot be reliable based on the given information. Screen time was transformed by rank-based normalization, and attention problems and internalizing problems phenotypes used the raw scores from the Child Behavior Checklist.

^b^ Children with clinically diagnosed attention-deficit/hyperactivity disorder and controls (those without such diagnosis) were used in the analysis.

^c^ Self-reported internalizing problems of children and adolescents aged 3 to 18 years were used as the phenotype.

^d^ Adult depression measured with broad self-reported questions was used as phenotype.

^e^ The parent-reported Mood and Feelings Questionnaire of children at 12 years of age was used as the phenotype.

^f^ The original source did not provide specific numbers of DZ and MZ twins.

^g^ Television time in adults was used as the phenotype.

^h^ Screen time of entertainment among adolescents aged 16 years was used as the phenotype.

**Figure 2.**
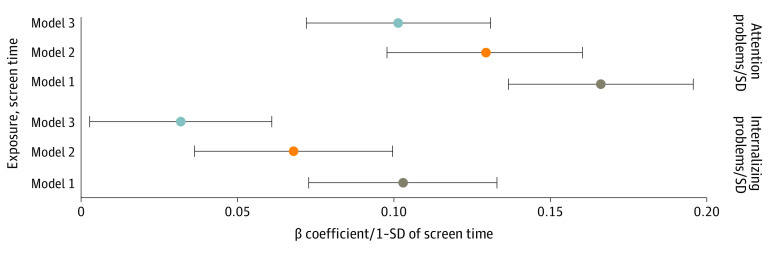
Associations of Child-Reported Screen Time With Parent-Reported Child Attention and Internalizing Problems (N = 4262) Model 1 was adjusted for sex and age. Model 2 was adjusted for sex, age, family income, and highest parental educational level. Model 3 was also adjusted for maternal psychopathological disorder. Screen time, attention problem, and internalizing problem scores were standardized to mean 0 and SD 1.

In fully adjusted models (model 3), each additional SD of child-reported screen time was associated with a 0.10-SD (95% CI, 0.07- to 0.13-SD) increase in attention problem score and a 0.03-SD (95% CI, 0.003- to 0.06-SD) increase in internalizing problem score (Figure 2). Similarly, each additional SD of parent-reported screen time was associated with a 0.10-SD (95% CI, 0.07- to 0.13-SD) increase in child attention problem score in a model adjusting for family income and highest parental educational level, but this estimate was attenuated and nonsignificant when also adjusting for maternal psychopathological disorder (β = 0.02; 95% CI, −0.01 to 0.05). A 1-SD higher parent-reported screen time was associated with a 0.05-SD (95% CI, 0.02- to 0.08-SD) increase in child internalizing problem score (eFigure 2 and eTable 5 in Supplement 1).

### Genetic Risk Scores 

**Figure 3.**
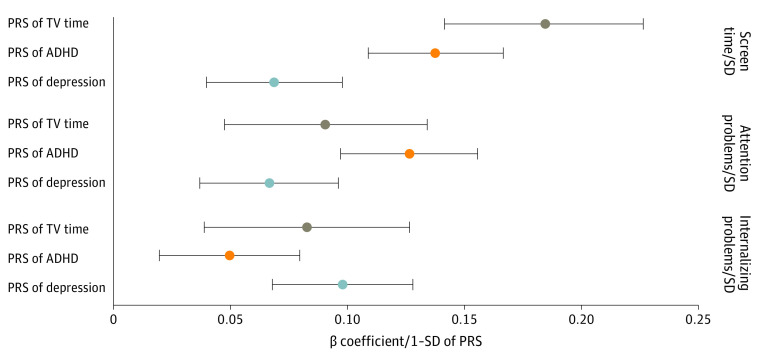
Associations of Polygenic Risk Score (PRS) With Screen Time, Attention Problems, and Internalizing Problems (N = 4262) Associations were adjusted for age, sex, study site, and top 10 principal components. Screen time, PRS, attention problems, and internalizing problems were standardized to mean 0 and SD 1. ADHD indicates attention-deficit/hyperactivity disorder; TV, television.

A 1-SD higher television time PRS was associated with a 0.18-SD (95% CI, 0.14- to 0.23-SD) longer child-reported screen time. The PRSs of ADHD (β = 0.14-SD; 95% CI, 0.11- to 0.17-SD) and depression (β = 0.07-SD; 95% CI, 0.04- to 0.10-SD) were, to a lesser extent, associated with screen time (Figure 3). Parent-reported child screen time was also associated with all 3 PRSs but with smaller magnitudes (eFigure 3 in Supplement 1).

A 1-SD higher ADHD PRS was associated with a 0.13-SD (95% CI, 0.10- to 0.16-SD) higher attention problem score. The PRSs of television (β = 0.09-SD (95% CI, 0.05-0.13) and depression (β = 0.07-SD (95% CI, 0.04-0.10) were, to a lesser extent, associated with attention problems (Figure 3). Likewise, higher PRSs were associated with more internalizing problems (Figure 3; eTable 6 in Supplement 1).

### Genetic Confounding

**Figure 4.**
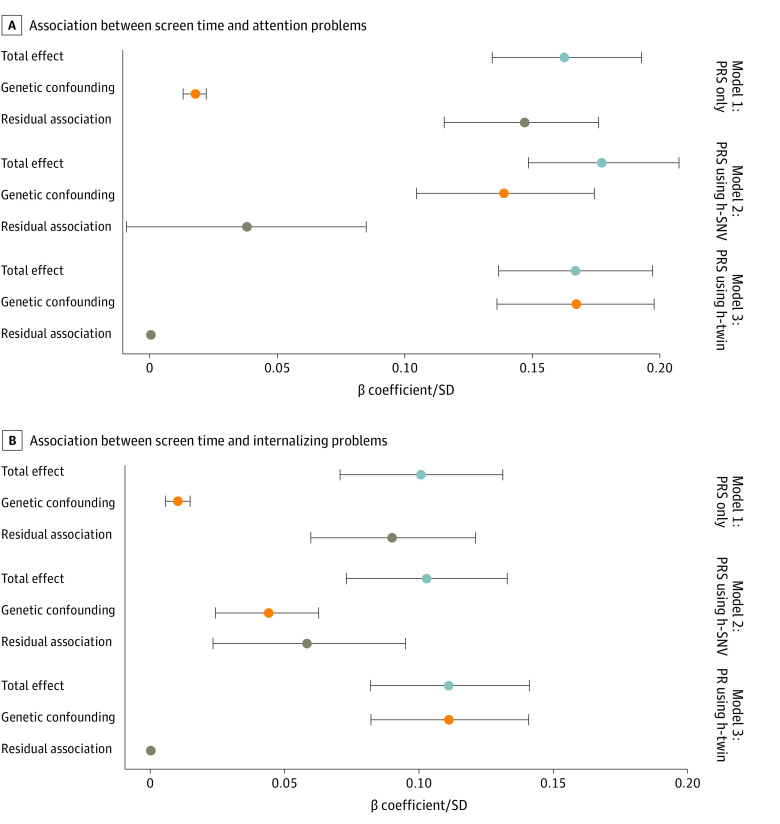
Association of Screen Time With Attention Problems and Internalizing Problems With Different Adjustments for Genetic Confounding (N = 4262) Model 1 was adjusted for polygenic risk scores (PRSs) for both exposure and outcomes only. Model 2 was adjusted for PRSs using single-nucleotide variant (SNV; formerly single-nucleotide polymorphism [SNP])–based heritability (h-SNV) for both exposure and outcomes. Model 3 was adjusted for PRSs using twin-based heritability (h-twin) for both exposure and outcomes.

In model 1, which was adjusted for both screen time and attention problem PRSs (not using SNV-based heritability or twin-based heritability), we found a small but significant genetic confounding (10.4%), indicating that approximately 10% of the phenotypic association between screen time and attention problems was explained by genetic confounding (Figure 4A). However, when the SNV-based heritability estimates for screen time and attention problems were used (model 2), the magnitude of genetic confounding was much larger, such that the association between screen time and attention problems was no longer statistically significant (Figure 4A). When twin-based heritability was used instead of SNV-based heritability (model 3), the results suggested that genetic confounding may entirely account for the association between child screen time and attention problems (Figure 4A).

Models of genetic confounding of the association between screen time and internalizing problems revealed similar patterns. Adjusting for PRSs only (model 1), we found a significant 10.0% genetic confounding for the association between screen time and internalizing problems (Figure 4B). Using the SNV-based heritability estimates (model 2), we observed a larger genetic confounding of 42.7% (Figure 4B). Using the twin-based heritability estimates (model 3), genetic confounding fully accounted for the screen time–internalizing problems association (Figure 4B; eTable 7 in Supplement 1).

The 2 most common subtypes of screen time (watching television or videos and playing video games) showed similar association patterns as total screen time, although genetic confounding affected the associations between watching television and psychiatric problems more so than it did playing video games (eFigure 4 in Supplement 1).

In sensitivity analyses that used models with only outcomes, genetic information suggested larger estimates of genetic confounding (eFigure 5 in Supplement 1). In other sensitivity analyses that adjusted for both genetic and socioeconomic factors (family income and highest parental educational level), we found similar percentages of genetic confounding, although the absolute effect sizes were reduced (eFigure 6 in Supplement 1). In sensitivity analyses that used parent-reported screen time, which may be affected by shared reporter bias, we found less obvious genetic confounding in the association between screen time and attention problems (45.8%) or internalizing problems (19.5%) in the model using PRS and SNV-based heritability (eFigure 7 in Supplement 1).

In sensitivity analyses that tested alternative plausible values of SNV-based heritability for screen time and psychiatric problems, the genetic confounding completely explained the association between screen time and attention problems when the attention problem SNV-based heritability was set to 0.24 (assuming the screen time SNV-based heritability was 0.08) (eFigure 8A in Supplement 1). Similarly, the residual association between screen time and internalizing problems disappeared when the SNV-based heritability of internalizing problems was set to 0.19 (eFigure 8B in Supplement 1).

## Discussion

This large population-based cohort study of screen time and child psychiatric problems in preadolescence suggests that genetic confounding, if modeled with genetic information from PRSs and heritability, may account for a substantial portion of the phenotypic association between screen time and child psychiatric problems. The study presented several findings. First, increased child screen time was associated with increased psychiatric problems, consistent with reports from prior research in the ABCD Study.^42^ This association was partially explained by sociodemographic factors and maternal psychopathological disorder but largely remained after the adjustments. Second, we found specificity in associations between PRSs and their corresponding traits, such as the television time PRS having higher association with child screen time compared with other traits. However, we also found associations between PRSs and other traits, such as the television time PRS being a factor in both attention and internalizing problems, suggesting horizontal pleiotropy of the genetic variants and thus possible genetic confounding (ie, a genetic predisposition that could be a factor in more screen time and psychiatric problems).

This study aimed to quantify genetic confounding in the association between child screen time and psychiatric problems using Gsens,^22^ a novel method that integrates information from both PRS and heritability estimates (either SNV-based heritability or twin-based heritability). The association between screen time and attention problems was highly confounded by genetic factors. In contrast, the association between screen time and internalizing problems was also genetically confounded but in a smaller magnitude. This residual association encompasses both the direct association of screen time with psychiatric problems and residual confounding by environmental factors (eg, parenting practices that may independently change both screen time and internalizing problems). Previous twin studies also similarly concluded that some lifestyle–behavior associations were largely explained by genetic confounding.^43,44,45^

The genetic confounding estimates in this study depended on the magnitudes of heritability estimates. Estimates for SNV-based heritability and twin-based heritability in the analytic sample were comparable with those of previous studies,^38,39,40,41^ despite differences in sample population, genetics quality control thresholds, and measurements (Table). With some fluctuations in the heritability estimates, the genetic confounding should be interpreted with caution. Although the study provides a range for potential genetic confounding based on SNV-based heritability and twin-based heritability, the true magnitude of genetic confounding may be less than the lower bound if SNV-based heritability was overestimated. Thus, the sensitivity analyses with a range of potential SNV-based heritability values can further contextualize the findings.

Some socioeconomic status measures may partly index genetic factors. For example, adjusting for maternal psychopathological disorder reduced the primary phenotypic associations possibly because mothers may transmit genetic variations that play a role in both child screen time and psychiatric problems. Furthermore, the proportion of genetic confounding remained comparable even after additionally adjusting the models for socioeconomic status. This finding suggests that adjusting for socioeconomic status captures some genetic confounding in the associations between screen time and psychiatric problems, consistent with previous reports that multiple genes are associated with both income and mental health.^46,47^

These results highlight the importance of considering genetic factors in sociobehavioral research. Considering genetic factors helps in better understanding complex causal associations. Many policymakers and scientists view child screen time as a modifiable risk factor. However, if genetic factors account for a large part of the observed association between screen time and mental health, then interventions restricting child screen time could be less effective in preventing child attention and internalizing problems than expected,^48^ consistent with similar findings on genetic confounding in the association between social media use and mental health.^49^ This finding does not suggest that parents should adopt a lenient attitude toward children who use electronic devices excessively, as increased screen time could be factors in other risks, such as reduced physical or academic activities.

### Limitations

This study has some limitations. First, the Gsens method assumed no gene-environment interactions. Yet, genetic confounding is likely to also exist in gene-environment interaction studies. Identifying genetic confounding could be insightful for both phenotypic and gene-environment interaction studies. Second, we cannot rule out reverse causality even though we assessed the outcome at the 1-year follow-up. It is possible that children with a genetic vulnerability for ADHD are more likely to be exposed to increased screen time.^50^ However, if so, the direction of the association between screen time and attention problems might be reversed, and the conclusion regarding genetic confounding would not change. Third, the PRSs in this analysis were derived from adult GWASs, which may make PRS-based genetic confounding estimates in children less precise. However, previous research has suggested a robust overlap of salient genetic factors between children and adults.^51^ Moreover, we used self-reported screen time in the absence of an objective measure, which can introduce differential measurement error and thus bias. However, the results obtained using parental report showed similar confounding patterns.

## Conclusions

Findings of this cohort study suggest that genetic confounding may account for much of the association between child screen time and attention problems and part of the association between screen time and internalizing problems. These results demonstrate the importance of considering genetic confounding using both PRSs and heritability information in sociobehavioral studies of modifiable factors for youth mental health.

## Notes

Supplement 1.
eMethods.
**eTable 1.** Correlation Matrix Among Child Reported Screen Time, Child Psychiatric Problems, and Polygenic Risk Scores (N=4262) **eFigure 1.** Original and Transformed Distribution of Child Reported Screen Time, Parent Reported Child Attention and Internalizing Problems **eTable 2.** Demographic Distribution of European Unrelated Individuals **eTable 3.** Demographic Distributions of European Children With Genetic Data Compared With European Children Without Genetic Data **eTable 4.** Heritability Estimates of Screen Subtypes and Corresponding Sample Sizes in Our Study **eFigure 2.** Comparison of Associations Between Child-Rated Versus Parent-Rated Reported Screen Time and Psychiatric Symptoms (N=4262) **eTable 5.** Associations of Child Screen Time With Attention and Internalizing Problems **eFigure 3.** Comparison of Associations Between Polygenic Scores (PRS) and Child-Rated Versus Parent-Rated Screen Time (N=4262) **eTable 6.** Associations of Polygenic Scores With Child-Reported Screen Time, Attention and Internalizing Problems **eFigure 4.** Association of Child Watching Television or Video Time and Playing Video Game Time With Attention Problems and Internalizing Problems: With Different Adjustments for Genetic Confounding (N=4262) **eFigure 5.** Association of Child Screen Time (Self-Reported) With Attention Problems and Internalizing Problems: With Different Adjustments for Genetic Confounding Using Genetic Information on the Outcome (GsensY) (N=4262) **eFigure 6.** Association of Child Screen Time (Self-Reported) With Attention Problems and Internalizing Problems: With Different Adjustments for Genetic Confounding and Additionally Residualizing on Socioeconomic Status (SES) (N=4262) **eFigure 7.** Association of Parent-Rated Child Screen Time With Attention Problems and Internalizing Problems: With Different Adjustments for Genetic Confounding (N=4262) **eFigure 8.** Sensitivity Analyses of Genetic Confounding Using Different SNP-based Heritability Simulated Values of ADHD in Two Scenarios of Screen Time Heritabilities **eTable 7.** Association of Screen Time With Attention Problems and Internalizing Problems: With Different Adjustments for Genetic Confounding, Additionally Adjusting for Socioeconomic Status

Supplement 2.
Data Sharing Statement
