# Supplementary material for: Shared Genetic Risk in the Association of Screen Time With Psychiatric Problems in Children
Source: JAMA Netw Open. 2023 Nov 6;6(11):e2341502. doi: 10.1001/jamanetworkopen.2023.41502 (PMC10628728; doi:10.1001/jamanetworkopen.2023.41502)
Supplement: Supplement 2. — Data Sharing Statement [file jamanetwopen-e2341502-s002.pdf]

Data Sharing Statement

Zhang. Shared Genetic Risk in the Association of Screen Time With Psychiatric Problems in Children. *JAMA Netw Open*. Published November 06, 2023. doi:10.1001/jamanetworkopen.2023.41502

Data
Data available: Yes
Data types: Data dictionary
How to access data: Adolescent Brain Cognitive Development is an open-access database and can be accessed at https://abcdstudy.org, held in the NIMH Data Archive. Data can be accessed following a data request to the NIH data access committee (https://nda.nih.gov/), which should include information on the planned topic of study. The following data sets were generated under NIMH Data Archive: The association of child screen time with psychiatric problems: the role of genetic confounding #2016 (https://doi.org/10.15154/1528687).
When available: With publication

Supporting Documents
Document types: None

Additional Information
Who can access the data: Anyone requesting the data.
Types of analyses: For any purpose.
Mechanisms of data availability: With investigator support.
